# Thyroid dysfunction induced by anti-PD-1 therapy is associated with a better progression-free survival in patients with advanced carcinoma

**DOI:** 10.1007/s00432-023-05364-z

**Published:** 2023-09-15

**Authors:** Yiran Lu, Qingchen Li, Lusi Xu, Yanqing Zheng, Yanchao Wang, Ying Liu, Rui Zhang, Lin Liao, Jianjun Dong

**Affiliations:** 1 Department of Endocrinology, Qilu Hospital, Shandong University, Ji-Nan, China; 2 Department of Medical Oncology, Qilu Hospital, Shandong University, Ji-Nan, China; 3 Department of Endocrinology and Metabology, Shandong Provincial Qianfoshan Hospital, Shandong University, Ji-Nan, China; 4 Department of Endocrinology and Metabology, The First Affiliated Hospital of Shandong First Medical University and Shandong Provincial Qianfoshan Hospital, Ji-Nan, China; 5 Department of Endocrinology and Metabology, Shandong Key Laboratory of Rheumatic Disease and Translational Medicine, The First Affiliated Hospital of Shandong First Medical University and Shandong Provincial Qianfoshan Hospital, Shandong Institute of Nephrology, Ji-Nan, China

**Keywords:** Thyroid dysfunction, Immune-related adverse events, Anti-programmed cell death 1(PD-1) therapy, Progression-free survival, Patients with advanced carcinoma

## Abstract

**Purpose:**

Thyroid dysfunction is the most common immune-related adverse event during anti-programmed cell death 1 (anti-PD-1) therapy. In this study, we monitored patients with advanced malignant tumors who received anti-PD-1 therapy to observe the characteristic of anti-PD-1 therapy-induced thyroid dysfunction and its correlation with prognosis.

**Methods:**

Patients with advanced carcinoma treated with anti-PD-1 therapy were evaluated for thyroid function at baseline and after treatment initiation from August 2020 to March 2022. Seventy-three patients were finally included in the study.

**Results:**

Among these patients, 19 (26.03%) developed thyroid dysfunction after receiving anti-PD-1 therapy. Primary hypothyroidism and thyrotoxicosis were the most common clinical manifestation. Anti-PD-1-induced thyroid dysfunction occurred 63 (26–131) days after administration; thyrotoxicosis appeared earlier than primary hypothyroidism. In Kaplan–Meier survival analysis, the progression-free survival (PFS) of the thyroid dysfunction group was better than that of the no thyroid dysfunction group (227 (95% confidence interval (CI) 50.85–403.15) days vs 164 (95% CI 77.76–250.24) days, *p* = 0.026). Male patients had better PFS than female patients (213 (95% CI 157.74–268.26) days vs 74 (95% CI 41.23–106.77) days, *p* = 0.031). In cox proportional hazards regression model, anti-PD-1-induced thyroid dysfunction remained an independent predictor of better PFS (hazard ratio (HR) = 0.339(0.136–0.848), *p* = 0.021).

**Conclusion:**

Thyroid dysfunction is a common immune-related adverse events in advanced cancer patients treated with anti-PD-1 therapy and predicts a better prognosis.

**Trial registration:**

This study was retrospectively registered with Trial ClinicalTrials.gov (NCT05593744) on October 25, 2022.

## Introduction

In recent years, the understanding of tumor immunological mechanisms has promoted the development of immune checkpoint inhibitors (ICIs) therapy. ICIs are monoclonal antibodies that target and regulate two key signaling pathways in T lymphocyte activation and exhaustion by binding to and inhibiting cytotoxic T lymphocyte antigen 4 or programmed cell death 1 (PD-1) and its ligand PD-L1, which can increase anti-tumor immune effect of T lymphocyte (Page et al. 2014; Topalian et al. 2016; Wright et al. 2021). As a landmark in tumor immunotherapy, ICIs have been approved for the treatment of a variety of malignant tumors, including melanoma, non-small cell lung cancer, esophageal cancer, gastric cancer, liver cancer, renal cancer, urothelial cancer and Hodgkin lymphoma (Chalan et al. 2018; Hargadon et al. 2018).

In addition to the anti-tumor effects, ICIs can cause organ-specific autoimmune responses, immune-related adverse events (irAEs), affecting a variety of organs including endocrine, lung, skin, gastrointestinal tract, etc. (Postow et al. 2018). Immune-related adverse events have attracted more attention with the increased application of ICIs. Although the cytological and molecular mechanisms that cause irAEs remain unclear, there is evidence that the type and severity of irAEs may be related to drugs and patients’ inherent characteristics (Khan and Gerber et al. 2020; Dougan et al. 2020).

Endocrine-related irAEs, such as thyroid dysfunction, hypophysitis, adrenal insufficiency and diabetes, are described with anti-PD-1 therapy (Wright et al. 2021). Most of these irAEs are mild and reversible if detected early and given appropriate treatment. But some can also lead to limited treatment or even threaten the patient’s life (Dougan et al. 2020).

Thyroid dysfunction, manifesting as hypothyroidism, thyrotoxicosis and thyroiditis, is the most common irAEs in the endocrine system, especially in anti-PD-1 therapy (Ferrari et al. 2018; Chang et al. 2019). Previous studies have shown that the incidence of ICIs therapy-induced thyroid dysfunction ranges from 21 to 54.41% (Osorio et al. 2017; Presotto et al. 2020; Thuillier et al. 2021; Paderi et al. 2021; Xu et al. 2022); however, there are few prospective studies in this area.

Although ICIs have improved the outcome of cancer therapy, their efficacy is still limited. Studies attempt to find biomarkers that can predict response to ICIs therapy. Several studies have demonstrated irAEs as potential clinical markers to predict response to ICIs therapy. However, the association between ICIs efficacy and the onset of ICIs-induced thyroid dysfunction is debated across the literature with conflicting results. Some studies have shown that the occurrence of ICIs-induced thyroid dysfunction predicts a better prognosis (Thuillier et al. 2021; Lima et al. 2021; Xu et al. 2022); however, some studies show that there is no significant correlation (Freeman-Keller et al. 2016; España et al. 2020; Muir et al. 2021; Rubino et al. 2021). In this study, we monitored patients with advanced malignant tumors who received anti-PD-1 therapy to observe the incidence of anti-PD-1 therapy-induced thyroid dysfunction and its correlation with prognosis.

## Methods

This was a prospective, observational and single-center study, conducted at East Branch of Qilu Hospital of Shandong University from August 2020 to March 2022.

### Population

Inclusion criteria were as follows: 1. aged 18 years or older; 2. confirmed diagnosis of malignancy by histologically or cytologically; 3. in stage III or IV according to the TNM staging and temporarily unable to undergo surgery; 4. received anti-PD-1 therapy. Exclusion criteria were as follows: 1. thyroid malignancy or history of thyroid malignancy; 2. thyroid dysfunction before anti-PD-1 therapy or receiving levothyroxine or antithyroid drugs; 3. previous ICIs treatment (including anti-PD-1/PD-L1(programmed death ligand 1) and anti-cytotoxic T lymphocyte antigen 4 treatment); 4. less than 2 cycles of anti-PD-1 treatment; 5. no thyroid function monitoring during anti-PD-1 treatment and missing data; 6. known pituitary disease; 7. pregnancy. This study was approved by the Ethics Committee of Qilu Hospital of Shandong University (KYLL-202208-042) and is registered with ClinicalTrials.gov (NCT 05593744). Written informed consent was provided by all participants.

The following patient characteristics were got from medical records: age, gender, height, weight, smoking history, alcohol use, diabetes history, hypertension history, tumor type, tumor stage, previous treatment, concomitant medication, etc.

### Treatment schedule and monitoring

Nivolumab (3 mg/kg) was administered by intravenous infusion every 2 weeks; sintilimab (200 mg), camrelizumab (200 mg), tislelizumab (200 mg) or pembrolizumab (200 mg) was administered by intravenous infusion every 3 weeks. Before and at least every two cycles during anti-PD-1 treatment, blood leukocytes, blood lymphocytes, blood hemoglobin, blood platelets, serum creatinine, serum alanine aminotransferase, serum aspartate aminotransferase, serum bilirubin and serum lactate dehydrogenase (LDH) were measured to evaluate safety of treatment. Tumor markers and X-ray computed tomography examinations were performed at least every 2 cycles to assess tumor status. Anti-PD-1 treatment continued until disease progression, death, occurrence of intolerable side effects, or withdrawal at the patient's discretion.

### Assessment

Thyroid function was detected before anti-PD-1 treatment, including serum-free triiodothyronine (fT3), free thyroxine (fT4), thyroid-stimulating hormone (TSH), or the patient's thyroid function was confirmed to be normal by thyroid function test in our hospital within half a year. Thyroid function (fT3, fT4 and TSH) was detected by chemiluminescence (Roche diagnostics GmbH, Germany) at least every 2 cycles during treatment; it was performed the day before anti-PD-1 treatment.

Considering the laboratory cutoffs, primary hypothyroidism was defined as isolated increased TSH levels with normal (subclinical form) or decreased (overt form) fT4. Thyrotoxicosis was defined as decreased TSH with normal (subclinical form) or increased (overt form) fT3 and/or fT4. Cases of a transient thyrotoxicosis followed by hypothyroidism were defined as biphasic thyroiditis, and they were not included in the events of primary hypothyroidism or thyrotoxicosis ^[16]^. Abnormal anti-thyroid antibody was defined as thyroglobulin antibodies and/or thyroid peroxidase antibodies exceeding the upper limit of the normal reference range. Lung immune prognostic index (LIPI) score was obtained according to serum LDH (lactate dehydrogenase) level and lymphocyte/neutrophil ratio (dNLR) (Mezquita et al. 2018). The prognosis was divided into 3 categories according to the LIPI score: good (normal LDH, dNLR < 3), intermediate (abnormal LDH or dNLR > 3), poor (abnormal LDH and dNLR > 3).

The clinical severity of thyroid dysfunction was graded using the Common Terminology Criteria for Adverse Events version 5.0. Progression-free survival (PFS) was defined as the time between initiation of anti-PD-1 therapy and disease progression or death, whichever came first. All follow-up ended on March 2022.

### Statistical analysis

Normally distributed continuous variables were expressed as mean ± standard deviation, and non-normally distributed continuous variables were expressed as median and interquartile range. Frequencies and percentages reported for categorical variables. The independent samples t test and nonparametric test (Mann–Whitney U test) were used to compare the differences between two groups of continuous variables; Chi-square test or corrected Chi-square test was used to study differences between categorical variables. Survival analysis was performed using the Kaplan–Meier method and compared by log-rank test. A Cox proportional hazard regression model was used for multivariable analysis to estimate the association between the occurrence of thyroid dysfunction and PFS, using variables if their *p* value is less than 0.2 in univariable analysis. All analyses were performed using IBM-SPSS version 21, and *p* value < 0.05 was considered statistically significant.

## Results

### Patient characteristics

**Fig. 1:**
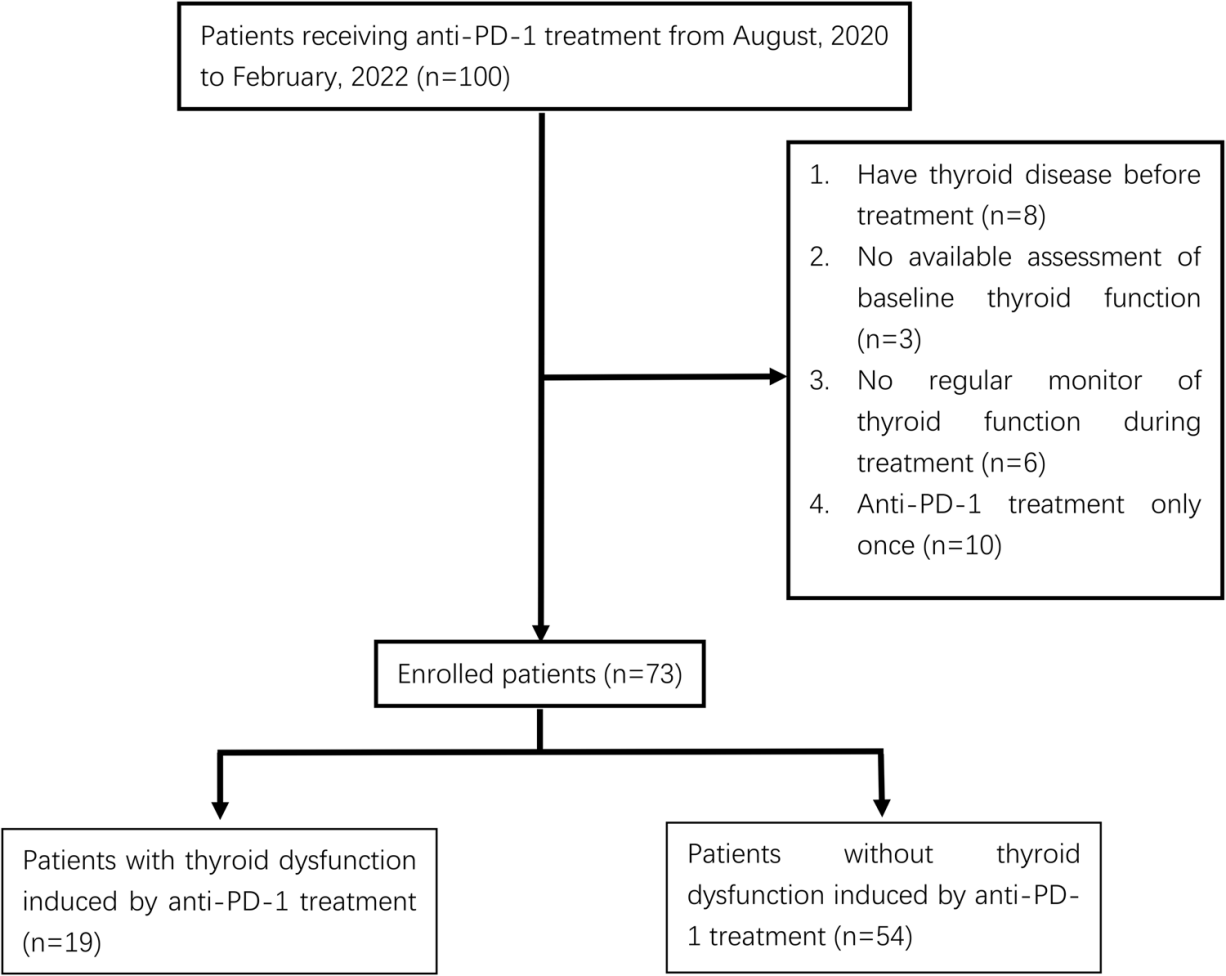
Flow chart of patients’ enrollment

A total of 100 patients received anti-PD-1 treatment in the Department of Oncology, East Branch of our hospital, during August 2020 to February 2022. Seventy-three patients were finally included in the study; the flowchart of the study is illustrated in Fig. 1.

The mean age of the 73 patients was 62.4 ± 10.5 years, included 59 males and 14 females. Among the 73 patients, 25 were gastric cancer, 18 were lung cancer, 14 were esophageal cancer, 4 were hepatocellular carcinoma, 3 were bladder urothelial carcinoma, 2 were colon cancer, 1 was poorly differentiated adenocarcinoma of the abdominal cavity (primary lesion unknown), 1 was Hodgkin's lymphoma, 1 was adrenocortical carcinoma, 1 was duodenal carcinoma, 1 was rectal cancer, 1 was ovarian cancer, and 1 was dual primary tumor of colon and stomach. Forty-six patients were treated with sintilimab, 17 with camrelizumab, 6 with tislelizumab, 3 received pembrolizumab, and 1 received nivolumab. Anti-PD-1 therapy was used in 35 patients as first-line therapy, 38 patients as second-line, or more than second-line therapy. Seven patients were treated as monotherapy, and 66 patients in combination with chemotherapy or targeted therapy.

Patients were grouped according to whether they have anti-PD-1 therapy-induced thyroid dysfunction, and the characteristics of patients are shown in Table 1. As shown in the table, there were no significant differences between the two groups in terms of age, gender, tumor type, tumor stage and baseline thyroid function. However, the treatment cycle and follow-up time in the thyroid dysfunction group were significantly higher than those in the no thyroid dysfunction group.

**Table 1:** Demographic characteristics of 73 patients received anti-PD-1 treatment

	Thyroid dysfunction (*n* = 19)	No thyroid dysfunction (*n* = 54)	*P* value
Age, years	61.2 ± 12.2	62.8 ± 10.0	0.564
Sex, *n* (%)			1.0
Male	15(78.9%)	44 (81.5%)	
Female	4 (20.1%)	10 (18.5%)	
Height, m	1.70 (1.65–1.70)	1.70 (1.64–1.73)	0.804
Weight, kg	66.6 ± 12.1	64.1 ± 10.1	0.398
BMI, kg/m^2^	23.3 ± 3.8	22.6 ± 3.2	0.534
Thyroid function at baseline			
fT3, pmol/L	4.26 (3.68–4.75)	4.45 (4.01–4.91)	0.425
fT4, pmol/L	14.5 (13–16.4)	15,64 (14.48–17.9)	0.101
TSH, uIU/ml	2.2 (1.74–2.87)	1.62 (1.21–2.32)	0.058
Primary neoplasm, *n* (%)			0.359
Gastric cancer	4 (21.1%)	21 (38.9%)	
Lung cancer	4 (21.1%)	14 (25.9%)	
Esophageal cancer	5 (26.3%)	9 (16.7%)	
Other cancers	6 (31.6%)	10 (18.5%)	
Disease stage, *n* (%)			0.673
III	5 (26.3%)	17 (31.5%)	
IV	14 (73.7%)	37 (68.5%)	
Anti-PD-1 agent, *n* (%)			0.092
Sintilimab	12 (63.2%)	34 (63%)	
Camrelizumab	2 (10.5%)	15 (27.8%)	
Other anti-PD-1 agents	5 (26.3%)	5 (9.3%)	
Alcohol use, *n* (%)			0.094
Yes	12 (63.2%)	22 (40.7%)	
No	7 (26.8%)	32 (59.3%)	
Smoking history, *n* (%)			
Yes	15 (78.9%)	31 (57.4%)	
No	4 (21.1%)	23 (42.6%)	
Combined chemotherapy or molecular targeted drugs, *n* (%)			0.771
Yes	18 (94.7%)	48 (88.9%)	
No	1 (5.3%)	6 (11.1%)	
Median numbers of treatment cycles, n	7 (4–18)	4 (2–6)	0.008*
Median follow-up period, days	130 (78–337)	71.5 (42–205.3)	0.022*

*BMI* body mass index, *fT3* free triiodothyronine, *fT4* free thyroxine; *TSH* thyroid-stimulating hormone

### Thyroid dysfunction

Among the 73 patients, 19 (26.03%) developed thyroid dysfunction after receiving anti-PD-1 therapy. Thyroid dysfunction in Patient No. 63 could not be classified (fT3 decreased, fT4 and TSH increased). The remaining 18 patients were classified according to thyroid function, including 11 patients with primary hypothyroidism, 4 patients with thyrotoxicosis and 3 patients with biphasic thyroiditis.

As shown in Table 2, anti-PD-1-induced thyroid dysfunction occurred 63 (26–131) days after administration. Thyrotoxicosis appeared earlier than primary hypothyroidism. According to the severity of irAEs, 16 patients were grade 1 when they first developed thyroid dysfunction. Three patients were grade 2 when they first developed thyroid dysfunction, requiring oral medication, but none discontinued immunotherapy because of the side effects. At the end of the follow-up, the thyroid function of 5 patients returned to normal. It took 20, 86, 24, 51 and 21 days, respectively, for thyroid dysfunction return to normal. Five patients needed levothyroxine supplementation due to clinical hypothyroidism. By the end of follow-up, they had been treated with levothyroxine for 141, 343, 277, 1 and 42 days, respectively. One patient needed metoprolol succinate to relieve palpitation symptoms due to thyrotoxicosis.

**Table 2:** Characterization of thyroid dysfunction events

	Primary hypothyroidism	Thyrotoxic	Biphasic thyroiditis	All thyroid dysfunction
Cases, *n* (%)	11/73 (15.07%)	4/73 (5.48%)	3/73 (4.11%)	19/73 (26.03%)
Age, years	61.5 ± 13.4	55.3 ± 13.9	68.3 ± 3.7	61.2 ± 12.2
Male, *n* (%)	9/11 (81.82%)	¾ (75%)	2/3 (66.67%)	15/19 (78.95%)
Time of onset, days	115 (46–185)	30.5 (17.5–57)	43 (23–66)	63 (26–131)
Severity	1–2	1–2	2	1–2

Among the 73 patients, 21 had the results of anti-thyroid antibody at baseline, and 5 of them had anti-thyroid antibody level above the normal range. The corrected Chi-square test showed that the presence or absence of abnormal anti-thyroid antibody at baseline was not associated with anti-PD-1 therapy-induced thyroid dysfunction during treatment (*p* = 0.856).

### Progression-free survival and its influencing factors

**Fig. 2:**
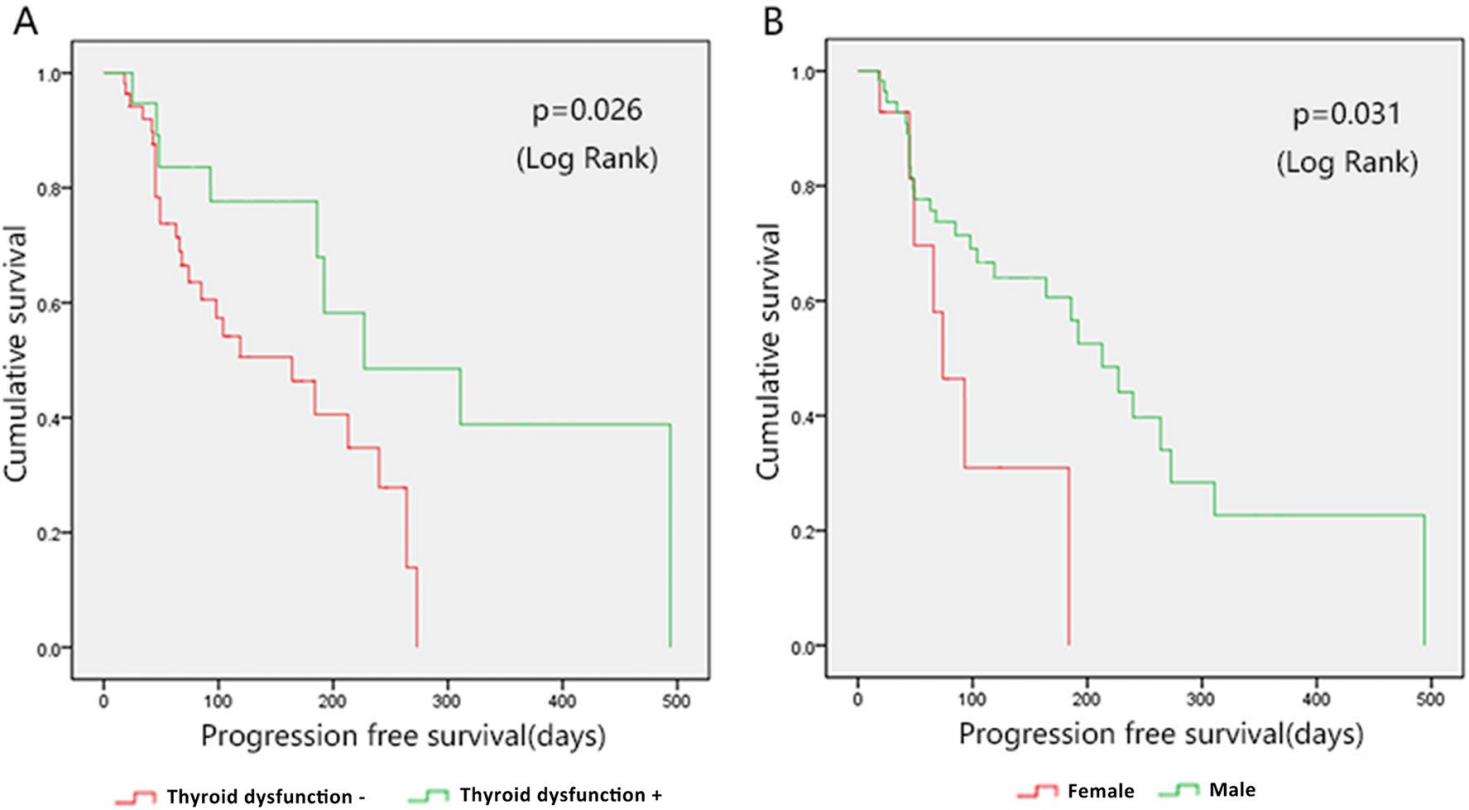
Survival analysis of patients receiving anti-PD-1 therapy. **A** PFS of patients with thyroid dysfunction and without thyroid dysfunction; **B** PFS of patients with different sex.

To explore the influencing factors of PFS, we performed survival analysis using Kaplan–Meier curve and log rank test. As shown in Fig. 2, the PFS of the thyroid dysfunction group was better than that of the no thyroid dysfunction group (227 (95% confidence interval (CI) 50.85–403.15) days vs 164 (95% CI 77.76–250.24) days, *p* = 0.026). Male patients had better PFS than female patients (213 (95% CI 157.74–268.26) days vs 74 (95% CI 41.23–106.77) days, *p* = 0.031). Age, tumor type, tumor stage, surgery history, radiotherapy history, diabetes, hypertension, smoking history, alcohol use, body mass index, anti-PD-1 drugs, first-line treatment or not, whether combined with chemotherapy or molecular targeted therapy, LIPI score and other effects on PFS were not statistically significant.

Factors with log rank test *p* < 0.2 in Kaplan–Meier survival analysis (thyroid dysfunction, gender, tumor stage, first-line treatment or not, LIPI; *p* values were 0.026, 0.031, 0.199, 0.141 and 0.091, respectively) were used to construct cox proportional hazards regression model. As shown in Table 3, anti-PD-1-induced thyroid dysfunction remained an independent predictor of better PFS (hazard ratio (HR) = 0.339 (0.136–0.848), *p* = 0.021).

**Table 3:** Cox proportional hazards regression modeling details

	Progression-free survival	
	Hazard ratio (95%CI)	P value
Thyroid dysfunction (yes vs no)	0.339 (0.136–0.848)	0.021*
Sex (male vs female)	0.609 (0.231–1.609)	0.317
Tumor stage (III vs IV)	0.647 (0.269–1.554)	0.330
First-line treatment (yes vs no)	0.455 (0.206–1.002)	0.051
LIPI score		
2 factors		0.189
1 factor	0.24 (0.049–1.177)	0.079
0 factor	0.336 (0.065–1.739)	0.194

*LIPI* lung immune prognostic index, *CI* confidence interval

## Discussion

Thyroid dysfunction is a common irAE in patients with malignancies treated with anti-PD-1 therapy. In this study, 26.03% of patients developed thyroid dysfunction during anti-PD-1 therapy, among which primary hypothyroidism was the most common type (15.07%). Hypothyroidism or thyrotoxicosis is the most common clinical manifestation of thyroid dysfunction induced by immunotherapy; sometimes, it is biphasic, with a transient thyrotoxicosis followed by hypothyroidism. Jeroen and colleagues further investigated the thyroid dysfunction by analyzing anti-thyroid antibodies and 18fluorodeoxyglucose-positron emission tomography/computed tomography images. The results showed that most of the patients with immunotherapy-induced thyroid dysfunction had elevated anti-thyroid antibodies and diffusely increased 18fluorodeoxyglucose uptake in thyroid, so it was considered that thyroid dysfunction was caused by destructive thyroiditis (Filette et al. 2016).

The vast majority of thyroid dysfunction occurred within six months after the initiation of anti-PD-1 therapy, and thyrotoxicosis appeared earlier than hypothyroidism. This is consistent with previous studies (Lima et al. 2021; Thuillier et al. 2021). Symptoms and signs of thyroid dysfunction are mostly mild, nonspecific and often masked by the patient's primary disease or side effects of other drugs. Thyroid dysfunctions were mostly grade 1–2 according to the severity of side effects in this study. Among them, patients with biphasic thyroiditis often required levothyroxine supplementation after the progression to hypothyroidism, which may be caused by serious destruction of the thyroid gland in the early stage. After early detection and symptomatic treatment, most patients' symptoms can be relieved, and no one in this study stopped anti-PD-1 therapy due to thyroid dysfunction. Therefore, early and accurate diagnosis and appropriate treatment of thyroid dysfunction are very important in clinical work. It is currently recommended to test thyroid function before the start of immunotherapy and every cycle for 3–6 months after initiation (Haanen et al. 2018; Castinetti et al. 2019).

The exact mechanism leading to immunotherapy-induced thyroid dysfunction remains unclear. The potential mechanisms include the breaking of immune tolerance, the cross reaction between tumor and normal tissues, and the aggravation of the patient’s pre-existing subclinical autoimmune state, which are related to the patient's genetic background and environmental factors (Boussiotis et al. 2016; Byrne et al. 2017; Pauken et al.^2019^; Dougan et al. 2020; Luo et al.^2021^). The most likely mechanism is that immunotherapy activates the immune system, which not only plays an anti-tumor effect, but also interferes with the immune tolerance of normal thyroid tissue (Álvarez-Sierra et al. 2019). T cells play an important role in the development of immunotherapy-induced thyroid dysfunction. The proportion of T cell in thyroid tissue specimens and peripheral circulation in patients with immunotherapy-induced thyroiditis was higher than that in the control group (Kotwal et al. 2020). Yasuda etc. have also confirmed that depletion of CD4 + T can completely prevent thyroid dysfunction caused by anti-PD-1 therapy, and depletion of CD8 + T cell can also partially prevent thyroid dysfunction caused by anti-PD-1 therapy in mice (Yasuda et al. 2021). Others such as natural killer cells and antigen-presenting cells may also be involved in the development of immunotherapy-induced thyroid dysfunction (Delivanis et al. 2017).

In the present study, baseline TSH levels were higher in the thyroid dysfunction group than in the no thyroid dysfunction group (although not statistically significant, *p* = 0.058). Previous studies have shown that elevated baseline TSH level was a risk factor for thyroid dysfunction in patients receiving nivolumab (Kimbara et al. 2018; Brilli et al. 2021). We speculate that patients with elevated baseline TSH level may inherently have low levels of thyroid autoimmunity and are therefore more likely to develop thyroid dysfunction after anti-PD-1 therapy. But this needs further research and verification. The study by Brilli et al. showed that if patients had a baseline TSH < 1.7mIU/L, thyroid dysfunction would not occur during immunotherapy and recommend that these patients do not need to monitor thyroid function during treatment (Brilli et al. 2021).

In this study, the presence of anti-thyroid antibodies (including thyroid peroxidase antibodies and thyroglobulin antibodies) at baseline was not associated with thyroid dysfunction during anti-PD-1 therapy. However, this may be due to the small sample size. Kimbara et al. found that the presence of anti-thyroid antibodies at baseline was a risk factor for thyroid dysfunction during nivolumab treatment (odds ratio = 9.19) (Kimbara et al. 2018). The study by Okada et al. also showed that patients with positive thyroid autoantibodies were much more likely to develop thyroid dysfunction during anti-PD-1 therapy than patients with negative thyroid autoantibodies (Okada et al. 2020). Yasuda et al. have also shown that the presence of autoimmune antibodies against thyroglobulin is essential for anti-PD-1-induced destructive thyroiditis in mice (Yasuda et al. 2021). It is unclear whether thyroid peroxidase antibodies and thyroglobulin antibodies have similar effects on the development of thyroid dysfunction in immunotherapy. It is generally believed that T cell-mediated cellular immunity is the most important cause of thyroid dysfunction induced by immunotherapy, and the above studies have shown that humoral immunity may also be involved in the process of irAEs. Its mechanisms need further study.

This study highlights that in patients with advanced malignancies receiving anti-PD-1 therapy, those with thyroid dysfunction have a better prognosis than those without. Xu et al. demonstrated that hypothyroidism predicts longer PFS in patients with hepatocellular carcinoma receiving anti-PD-1 therapy (Xu et al. 2022). Thuillier et al. also showed that in patients with non-small cell lung cancer, thyroid dysfunction induced by nivolumab also predicted longer overall survival and PFS (Thuillier et al. 2021). Our study suggests that thyroid dysfunction induced by anti-PD-1 therapy may be a clinical marker of better prognosis in different types of tumors.

In this study, there was no difference in the probability of developing thyroid dysfunction between patients of different genders receiving anti-PD-1 therapy. Survival analysis showed that male patients had longer PFS when receiving anti-PD-1 therapy, which was consistent with previous studies (Conforti et al.^2018^). Sex hormones may play a role in modulating the effects of immunotherapy. Studies have shown that both estrogen and androgen can bind to specific receptors on immune cells to regulate the function of the immune system. It is generally believed that estrogens enhance the activity of the immune system, while androgens are immunosuppressive (Moulton 2018). This also leads to differences in the immune response between males and females, resulting in different incidences of some diseases in different genders, such as Graves' disease and Hashimoto's thyroiditis, both of which occur more frequently in women. Studies have shown that sex hormones can affect the expression of PD-1 on T lymphocytes, macrophages, B lymphocytes, dendritic cells and other immune cells, but the specific mechanism of gender differences affecting the effect of immunotherapy needs further research (Dinesh et al. 2010).

LIPI score is derived from serum lactate dehydrogenase level and lymphocyte/neutrophil ratio, which can be used to predict the prognosis of patients with non-small cell lung cancer receiving immunotherapy, chemotherapy, and targeted therapy (Mezquita et al.^2018^; Kazandjian et al.^2019^). Studies have shown that LIPI score can also predict the prognosis of immunotherapy and chemotherapy in patients with other solid tumors (Meyers et al.^2019^; Liu et al.^2021^; Pan et al.^2022^). In this study, the LIPI score could not predict the prognosis of patients with advanced tumor receiving anti-PD-1 therapy, which may be due to the small sample size.

Our study has the following limitations. (A) Due to policy changed caused by Corona Virus Disease 2019, study follow-up ended in March 2022, resulting in some patients not receiving follow-up on disease progression or death. (B) As it was a single-center study, the sample size was insufficient for subgroup analysis of different types of thyroid dysfunction. (C) Fewer patients were detected for anti-thyroid antibodies. Whether anti-thyroid antibodies are related to anti-PD-1 therapy-induced thyroid dysfunction requires multi-center studies with larger sample size to verify.

## Conclusions

Thyroid dysfunction is a common immune-related adverse events in advanced cancer patients treated with anti-PD-1 therapy; primary hypothyroidism and thyrotoxicosis are the most common manifestations. Thyroid dysfunction developed during anti-PD-1 therapy predicts a better prognosis. Regular detection of thyroid function during anti-PD-1 therapies, early diagnosis and appropriate treatment to prevent severe symptoms affecting the treatment of the primary disease is very important.

## Data Availability

De-identified individual data might be available following publication by reasonable request to the corresponding author accompanied by research proposal.
